# Seed Coat Microsculpturing Is Related to Genomic Components in Wild *Brassica juncea* and *Sinapis arvensis*

**DOI:** 10.1371/journal.pone.0083634

**Published:** 2013-12-30

**Authors:** Ying-hao Wang, Wei Wei, Ding-ming Kang, Ke-ping Ma

**Affiliations:** 1 College of Agriculture and Biotechnology, China Agricultural University, Beijing, China; 2 State Key Laboratory of Vegetation and Environmental Change, Institute of Botany, Chinese Academy of Sciences, Beijing, China; New Mexico State University, United States of America

## Abstract

It has been reported that wild *Brassica* and related species are widely distributed across Xinjiang, China, and there has been an argument for species identification. Seed coat microsculpturing (SCM) is known to be an excellent character for taxonomic and evolutionary studies. By identifying collections from Xinjiang, China, and combining SCM pattern, flow cytometry, and genome-specific DNA markers as well as sexual compatibility with known species, this study aimed to detect potential relationships between SCM and genomic types in wild *Brassica* and related species. Three wild collections were found to be tetraploid with a SCM reticulate pattern similar to *B. juncea*, and containing A and B genome-specific loci, indicating relatively high sexual compatibility with *B. juncea.* The others were diploid, carrying S-genome-specific DNA markers, and having relatively high sexual compatibility with *Sinapis arvensis.* Moreover, their SCM was in a rugose pattern similar to that of *S. arvensis*. It was suggested that SCM, as a morphological characteristic, can reflect genomic type, and be used to distinguish B-genome species such as *B. juncea* from the related *S. arvensis*. The relationship between SCM and genomic type can support taxonomic studies of the wild *Brassica* species and related species.

## Introduction

The Brassicaceae family comprises about 338 genera and 3700 species, the majority of which are distributed in the temperate areas of the Northern Hemisphere [1]. *Brassica* species have great economic significance for agriculture because of their value as oil crops or vegetables. Recent reviews, including Warwick and Black [2], have described the cytological and taxonomic relationships of *Brassica* and related species. A commonly agreed pattern describes species relationships within the genera *Brassica* is the U-triangle, which classified genome types into diploids AA (*Brassica rapa*), BB (*B. nigra*), and CC (*B. oleracea*) and their allopolyploids AABB (*B. juncea*), AACC (*B. napus*), and BBCC (*B. carinata*) [3].

The seed coat exists at the interface between the embryo and the exterior environment, promoting seed dispersal, survival in adverse environments, and protection from pests and pathogens. Based on morphological and anatomical studies, seed coat morphology observed under the scanning electron microscope (SEM) is a reliable approach to taxonomic and evolutionary analysis [4]–[8]. The terminologies of seed coat microsculpturing (SCM) are easy to understand, and more importantly, the descriptions are in agreement with the various seed samples under study [8]–[10]. The seed coat patterns in amphidiploid *Brassica* species exhibit either an intermediate seed coat pattern between the two putative diploid parents, or only one of the two ancestral parents [8]. While a large body of research describes the anatomy of seed coats of Brassicaceae plants, to date only a few wild species have been observed [10]–[13]. Collectively these studies suggest that SCM patterns at high magnification are species-specific in *Brassica* and its related species. However, no other evidence exists to confirm this hypothesis, and no study has taken into consideration the relationship between SCM and plant genomic types. A large number of genome specific genetic markers have been developed, in particular for *Brassica* and related species in Brassicaceae, and these can be used to verify the SCM pattern of individual species.

Crop wild relatives and their diversity are widely held to be the most important component of plant genetic resources used for developing new cultivars in agriculture [14], and wild *Brassica*s and related species can contribute useful traits for crop breeding. Xinjiang Uighur Autonomous Region is located in the northwestern border of China and in the hinterland of the Eurasian Continent, where wide distribution of wild *Brassica* and related species has been frequently reported [15]–[17]. In this study, wild *Brassica* species and morphologically similar plants were collected from this region and species identification and the role of SCM patterns was investigated by scanning electron microscopy, combining genome specific DNA markers, flow cytometry, and the sexual compatibility of hybridization with known species. Our results add useful information for the wild germplasm resources of *Brassica* crops, provide a practical methodology for plant identification in *Brassica* and related species, and contribute to the understanding of the possible relationship(s) between SCM patterns and genomic types.

## Materials and Methods

### Plant Materials

Two sets of wild collections of *Brassica* and related species were investigated in this study and three species (*B. juncea*, *B. nigra* and *Sinapis arvensis*) were used as controls (Table 1). One set of 12 accession numbers was kindly provided by the Xinjiang (XJ) Agricultural Academy and named XJ-4, XJ-5, XJ-6, XJ-7, XJ-8, XJ-9, XJ-10, XJ-11, XJ-12, XJ-13, XJ-14 and XJ-Baicheng. Another set of 13 accessions was collected in 2011 by us in Zhaosu (ZS), Xinjiang, China and named as ZS-2, ZS-4, ZS-5, ZS-6, ZS-7, ZS-8, ZS-9, ZS-10, ZS-11, ZS-13, ZS-14, ZS-15, and ZS-16. The seeds were grown in the greenhouse of the Institute of Botany, Chinese Academy of Sciences, Beijing, China, during 2011 and 2012. Two more accession numbers, *B. campestris* and *B. oleracea*, were used as controls during scanning electron microscopy analysis of SCM pattern.

**Table 1:** List of accessions studied for seed coat microsculpturing pattern.

Species	No. of accessions	Common name	Ploidy level	Genome	Place ofcollection/origin	Source
***B. campestris***	1	Green Chinese cabbage	2×	AA (n = 10)	Hongkong, China	Supplied by Oilcrops Research Institute, Chinese Academy of Agricultural Sciences, China
***B. nigra***	1	Black mustard	2×	BB (n = 8)	Australia	Supplied by Beijing Vegetable Center, China
***B. nigra***	1	Black mustard	2×	BB (n = 8)	Crucifer Genetics Cooperative	Supplied by Beijing Vegetable Center, China
***B. nigra***	1	Black mustard	2×	BB (n = 8)	Russia	Supplied by Beijing Vegetable Center, China
***B. oleracea***	1	Cabbage	2×	CC (n = 9)	Wuhan, Hubei,China	Supplied by Oilcrops Research Institute, Chinese Academy of Agricultural Sciences, China
***S. arvensis***	1	Wild mustard	2×	SS (n = 18)	Canada	Supplied by Agriculture and Agri-Food Canada (AAFC), Canada
***S. arvensis***	1	Wild mustard	2×	SS (n = 18)	France-1	Supplied by INRA, France
***S. arvensis***	1	Wild mustard	2×	SS (n = 18)	France-2	Supplied by INRA, France
***B. napus***	1	Canola	4×	AACC (n = 19)	Canada	Supplied by University of Tennessee, USA
***B. juncea***	1	Weedy brown mustard	4×	AABB (n = 18)	Xining, Qinghai, China	Supplied by Qinghai Academy of Agricultural Sciences, China
***B. juncea***	1	Weedy brown mustard	4×	AABB (n = 18)	Nanjing, Jiangsu, China	Supplied by Nanjing Agricultural University, China
**Wild ** ***collection***	13	Xinjiang wild rape	–	–	Zhaosu (ZS), Xinjiang,China	Collected in the field by the authors in 2011
**Wild ** ***collection***	12	Xinjiang wild rape	–	–	Xinjiang (XJ), China	Supplied by Xinjiang Academy of Agricultural Sciences (XJ), China

### Stereoscopic Microscope and Scanning Electron Microscopy Observation

The materials used for stereoscopic microscope and scanning electron microscopy (SEM) observation were dried mature seeds. At least three seeds of each accession number were chosen as representatives for scanning electron microscopy (SEM) studies. The seeds were dehydrated through a graduated ethanol series and fixed on aluminum stubs by using double-sided adhesive and coated with a thin film of silver (Hitachi ion sputter coater E-1010, Japan). SEM was performed using a Hitachi-S4800 FESEM (field emission scanning electron microscope, Tokyo, Japan) at 10 kV at low magnification (×50) and higher magnification (×250, ×800), respectively, and scanned photos of seed coat microsculpturing (SCM) were subjected to comparative analysis.

### Molecular Identification

Genomic DNA was isolated from young leaves using a DNAsecure Plant DNA Kit (Tiangen Co., China). The following specific polymorphic DNA markers were selected for the A, B, C and S genomes (Supporting Information, Table S1): (1) simple sequence repeats (SSRs) (Na10-B01, Na10-D09, Ni2-E04, BN83B1 and Na12-C08) [18]–[21]; (2) B-genome-specific primer (pBNBH35) [22]; and (3) sequence characterized amplified regions (SCAR) found in both B-genome *Brassica* species and *S. arvensis* (COL1.1, SLR1.1, LFYa.5) [23]. In brief, these primers included one pair specific to the A genome, two pairs for the B genome, two pairs for the C genome, one pair for the A and B genomes, and three pairs for the B and S genomes. *B. nigra*, *B. juncea*, *B. napus*, and *S. arvensis* were used as control species.

The polymerase chain reaction (PCR) for these DNA markers was performed using 2×Taq PCR MasterMix (Tiangen Co., China). The reaction mixture was initially denatured at 94°C for 5 min, followed by 35 cycles of amplification at 94°C for 1 min, at PCR annealing temperature (Tm, °C ) for 1 min or 30 s, and 72°C for 1 min, and a final extension at 72°C for 7 min. The Tm varied among different DNA markers (Table S1). The PCR products were fractionated on a 1.5% agarose gel. After electrophoresis, the gel was viewed with a UV illuminator.

### Flow Cytometry Estimation of Plant Ploidy

Flow cytometry (FCM) is commonly used to estimate plant genome size among species, and to differentiate plant ploidy levels in various tissues. A common method [24] was used to release nuclei in this study. Plant leaves were chopped using a sharp razor blade in a dish with Galbraith’s buffer (45 mM MgCl_2_, 20 mM MOPS, 30 mM sodium citrate, 0.1% (vol/vol) Triton X-100). The pH value was adjusted to 7.0 with 1 M NaOH, and the solution was filtered through a 0.22 mm filter and stored at –20°C in 10 ml aliquots. The isolated nuclei were then stained with a DNA fluorochrome, 40, 6-diamidino-2-phenylindole (DAPI, stock solution 0.1 mg ml^–1^). Following filtering through a 0.22 mm filter to remove small particles, the solution was stored at –20°C in 1 ml aliquots, and subjected to flow cytometric measurement using a FCM machine (Beckman Coulter MoFlo XDP). Reference standards for direct DNA estimation were *S. arvensis* (France-1) as diploid and *B. napus* (Canada) and *B. juncea* (Xining, China) as tetraploid (Table 1). Three wild accession numbers (ZS-5, ZS-11, and ZS-13) that represented the genomic types of wild collections in this study were subjected for FCM ploidy analysis. Each accession was repeated for at least five individual plants. FCM analysis of DNA ploidy level showed histograms with defined peaks. The mean channel number (mean fluorescence light, FL) and coefficient of variation value (CV, %) of each peak was calculated. Results with percentage of coefficient variation below 3% were considered reliable, and those with more than 5% were considered unacceptable. The ploidy of the unknown sample was calculated using the mean FL of the G_1_ peak in sample and reference [24]:
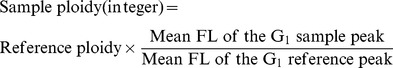



### Interspecific Hybridization

Wild collections ZS-11 and ZS-13 proved to be distinct from the others. One of the others (ZS-5) was chosen, together with ZS-11 and ZS-13, to hybridize with known species, i.e. *B. nigra* (Australia), *B. juncea* (Xining, China) and *S. arvensis* (France-1), for further identification. Seeds were sown in the greenhouse, and upon flowering, at least five individual plants of each of the three wild *Brassica* collections were emasculated and pollinated with pollens from more than five individual plants of each species of *B. nigra*, *B. juncea*, or *S. arvensis* to obtain potential hybrid progeny. Each of the three paternal species were self-pollinated among three plants of the same species and used as controls. At least 50 flowers per plant were crossed, with the exception that a lower number of flowers per plant were pollinated in the crossing with *B. nigra* due to limited flowering on the plants.

One plant of each wild collection number was also pollinated by known species, i.e. *B. nigra* (Australia), *B. juncea* (Xining, China) and *S. arvensis* (France-1), to generate hybrid seeds for SCM scanning. Some pollinated wild plants produced no seed (data not shown). As a significant distinction in SCM patterns were observed between *Brassica* plants (especially the B-genome species e.g. *B. juncea* and *B. nigra*) and *S. arvensis*, which were easily observed using SEM technology, reciprocal hand-crossing was also conducted between *B. juncea* and *S. arvensis*. The hybrid F_1_ formed between *B. juncea* (Xining, China) and *S. arvensis* (France-1) was analyzed and then backcrossed to obtain backcrossing progenies BC_1_-1 with the paternal species and BC_1_-2 with the maternal plant, respectively. More than four individual plants were used for each of parents during crossing and backcrossing between *B. juncea* and *S. arvensis*. All the hybrids and backcrossed progenies were subjected to SCM scanning by electron microscopy.

Hybridization is a traditional and direct method for identifying species. The number of pollinated flowers per plant, number of pods set per plant, percentage of pod setting (%), number of mature seeds per pod, and sexual compatibility index in different cross combinations were recorded, calculated, and subjected to analysis of variance (ANOVA). Difference was compared by the Scheffe’s test for unequal numbers of replications. All statistics were run using SPSS version 16.0 (SPSS Inc., 2008). The significant level was set at 0.05. The percentage of pod setting (%) was calculated as the percentage ratio between the mean number of full pods and flowers by hand cross-pollination. The sexual compatibility index was calculated as the ratio of the mean number of sets of full seeds to the number of pollinated flowers by hand crossing.

## Results

### Seed Coat Microsculpturing (SCM) Pattern of the Wild *Brassica* Species

**Figure 1:**
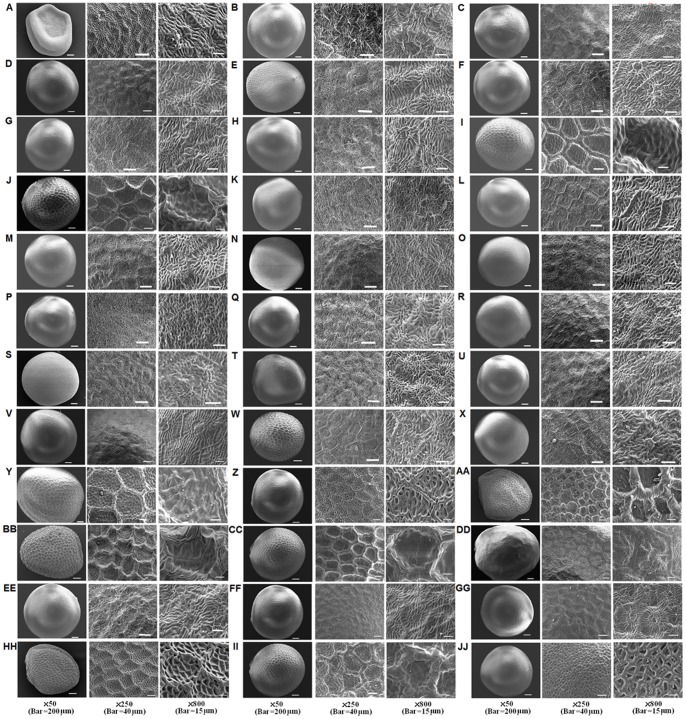
Scanning electron micrographs of seed coat microsculpturing patterns. (A)ZS-2, (B) ZS-4, (C) ZS-5, (D) ZS-6, (E)ZS-7, (F)ZS-8, (G)ZS-9, (H)ZS-10, (I)ZS-11, (J)ZS-13, (K)ZS-14, (L)ZS-15, (M)ZS-16, (N)XJ-4, (O)XJ-5, (P)XJ-6, (Q)XJ-7, (R)XJ-8, (S)XJ-9, (T)XJ-10, (U)XJ-11, (V)XJ-12, (W)XJ-13, (X)XJ-14, (Y) XJ-Baicheng, (Z) *B. campestris* (Hongkong, China), (AA) *B. nigra*(Australia), (BB) *B. nigra* (CRGC), (CC) *B. nigra* (Russia), (DD) *B. oleracea* (Wuhan, China), (EE) *S. arvensis* (France-1), (FF) *S. arvensis* (Canada), (GG) *S. arvensis* (France-2), (HH) *B. juncea* (Nanjing, China), (II) *B. juncea* (Xining, China), and (JJ) *B. napus* (Canada).

Seeds were round plump or shriveled and sized between 1.2 to 2 mm×1.1 to 1.7 mm. Seed coat color was tan, brown, dark brown, or yellow. Representative scanning electron microscopy (SEM) patterns of seed coat microsculpturing (SCM) are visible at lower (×50) and higher magnifications (×250, ×800), respectively (Fig. 1). For *B. nigra,* all three accessions had a reticulate SCM pattern of a raised network presenting a geometric appearance, and each area was outlined by a high, and wide reticulum wall, and a flat and smooth interspace. In one accession (CRGC), the reticulum interspace contained slight undulations. Both accessions of *B. juncea* had a reticulate pattern, but varied markedly in the nature of the reticulum. In one accession (Xining, China), the reticulum interspace contained undulations traversing the interspace, with a high and wide reticulum wall. In the other accession number (Nanjing), the reticulum interspace contained small daughter reticulations, with a low and thin reticulum wall. *B. napus* was in a foveate pattern having irregular and dense reticulum with small pits. All three accession numbers of *S. arvensis* had a rugose pattern, the irregular elevations making up the wrinkles and running mostly in one direction. *B. campestris* had an irregular reticulate-foveate pattern of a type intermediate between reticulate and foveate types, having a low and narrow reticulum wall and a reticulum interspace with small pits. *B. oleracea* had a reticulate-rugose pattern of a type intermediate between reticulate and rugose types (Fig. 1).

The SCM of the plant materials examined in this study can be basically divided into two types: (1) a reticulate type (*B. campestris*, *B. juncea*, *B. nigra*, *B. oleracea* and three wild accessions (ZS-11, ZS-13 and XJ-Baicheng)); and (2) a rugose type with a wave like pattern (*S. arvensis*, and the other wild accessions). The seed coat SCM patterns of ZS-11, ZS-13, and XJ-Baicheng were similar to *B. nigra* (Russia) *and B. juncea* (Xining, China), namely a reticulate pattern with a high and wide reticulum wall, and the reticulum interspace containing undulations traversing the interspace. XJ-Baicheng had larger reticulum than the others. The other wild accession numbers were similar to *S. arvensis* with respect to rugose pattern.

The results showed that SCM patterns of the species with B genome (e.g. *B. nigra* and *B. juncea*) had a reticulate pattern, while the plants of *S. arvensis* that contained the S-genome appeared with a rugose seed coat pattern. Seeds of ZS-11, ZS-13, and XJ-Baicheng had a similar SCM pattern to the B-genome species, while the other wild accessions resembled the S genome species (*S. arvensis*). In addition, the SCM pattern of hybrid seeds was found to be the similar to their maternal parent; i.e., reticulate when the maternal plant was B-genomic similar species, and rugose when the maternal plant was S-genomic similar species (Supporting Information, Fig. S1). For instance, when XJ-Baicheng was used as the maternal parent, the hybrid seeds showed a reticulate pattern, irrespective of whether a B-genome or S-genome species was used as the pollen donor (Fig. S1).

**Figure 2:**
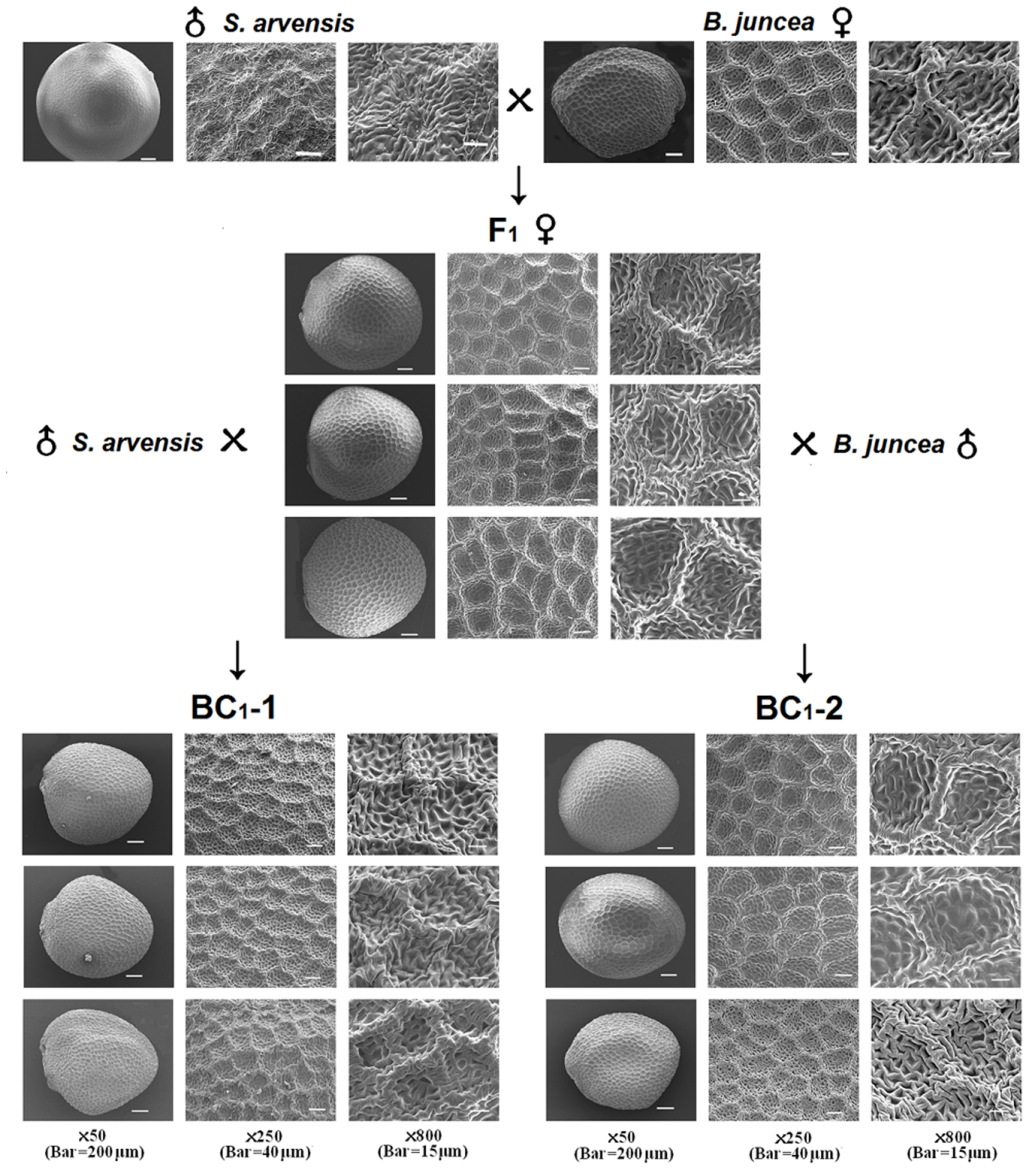
Scanning electron micrographs of seed coat microsculpturing patterns in hybrid and backcrossed seeds formed between *B. juncea* (Xining, China) and *S. arvensis* (France-1).

The SCM of the reciprocal crossed progenies between *B. juncea* and *S. arvensis* had a reticulate pattern (Fig. 2) similar to the maternal parent in F_1_, BC_1_-1, and BC_1_-2 although there should be genetic segregation in the backcross. The hybridization status was confirmed by flow cytometry, showing the ploidy level in F_1_ hybrids intermediated between their two parents (*B. juncea* and *S. arvensis*) (Supporting Information, Fig. S2). These results demonstrate that the SCM pattern of seed coats could be defined by maternal effect.

### Molecular Identification

**Figure 3:**
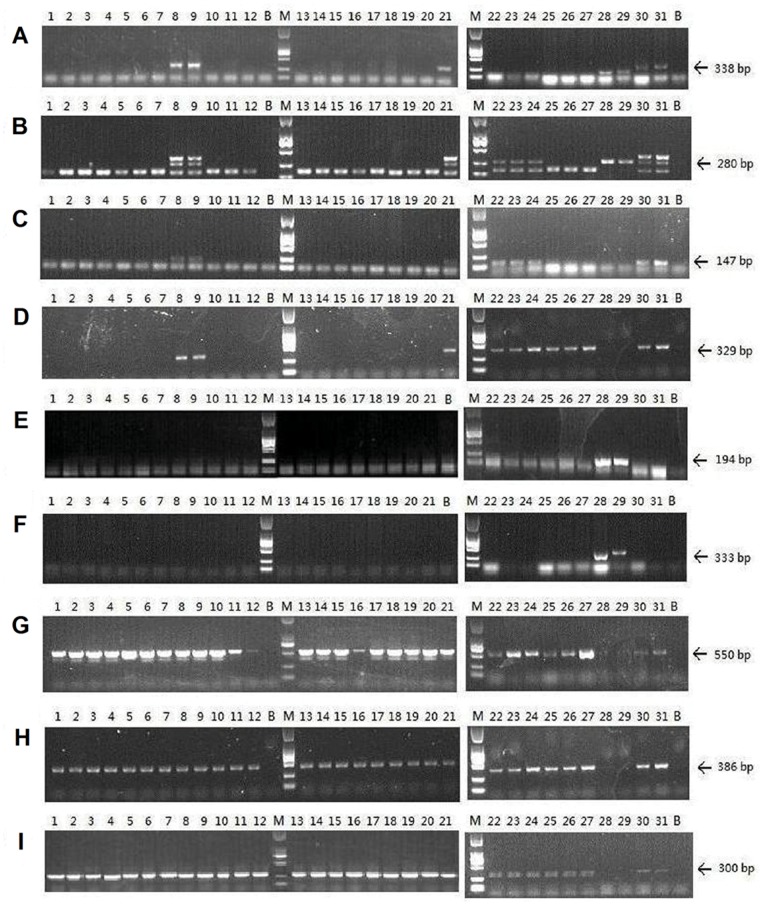
DNA amplification of specific primers separated by a 1.5% agarose gel. (A) Na10-B01, (B) Na10-D09, (C) Ni2-E04, (D) pBNBH35, (E) BN83B1, (F) Na12-C08, (G) COL1.1, (H) SLR1.1, and (I) LFYa.5. 1: ZS-4, 2: ZS-5, 3: ZS-6, 4: ZS-7, 5: ZS-8, 6: ZS-9, 7:ZS-10, 8: ZS-11, 9: ZS-13, 10: ZS-14, 11: ZS-15, 12: ZS-16, 13: XJ-4, 14 : XJ-7, 15: XJ-9, 16 : XJ-10, 17: XJ-11, 18 : XJ-12, 19: XJ-13, 20: XJ-14, 21: XJ-Baicheng, 22: *B. nigra* (Australia), 23: *B. nigra* (CRGC), 24: *B. nigra* (Russia), 25: *S. avensis* (Canada), 26: *S. avensis* (France-1), 27: *S. avensis* (France-2), 28: *B. napus* (Canada), 29: *B. napus* (Canada), 30: *B. juncea* (Nanjing, China), 31: *B. juncea* (Xining, China), B: Blank control, M: Marker DL-2000.

Using genomic DNA extracted from the wild collections and control species, the primer specific to the A genome (Na10-B01) amplified a 338 bp fragment in the PCR reaction. The two primer pairs specific to the B genome (Ni2-E04 and pBNBH35) amplified fragments of 147 bp and 329 bp, respectively. Amplified C genome specific fragments for primer BN83B1 and Na12-C08 were 194 bp and 333 bp, respectively. The PCR fragment amplified from Na10-D09 resolved as a unique single band on agarose gel at a size of 280 bp for the A and B genomes. The three primer pairs (COL1.1, SLR1.1 and LFYa.5) for the B and S genomes produced 550 bp, 386 bp, and 300 bp bands on agarose gel, respectively. The wild accession numbers of ZS-11, SZ-13, and XJ-Baicheng produced A and B genome specific fragments, while the other wild accession numbers produced S genome specific products (Fig. 3). These results suggested that ZS-11, ZS-13 and XJ-Baicheng contains both A and B genomes and could be *B. juncea*, while the other wild accessions contained the S genome and could be *S. arvensis*.

### Flow Cytometry Identification

**Figure 4:**
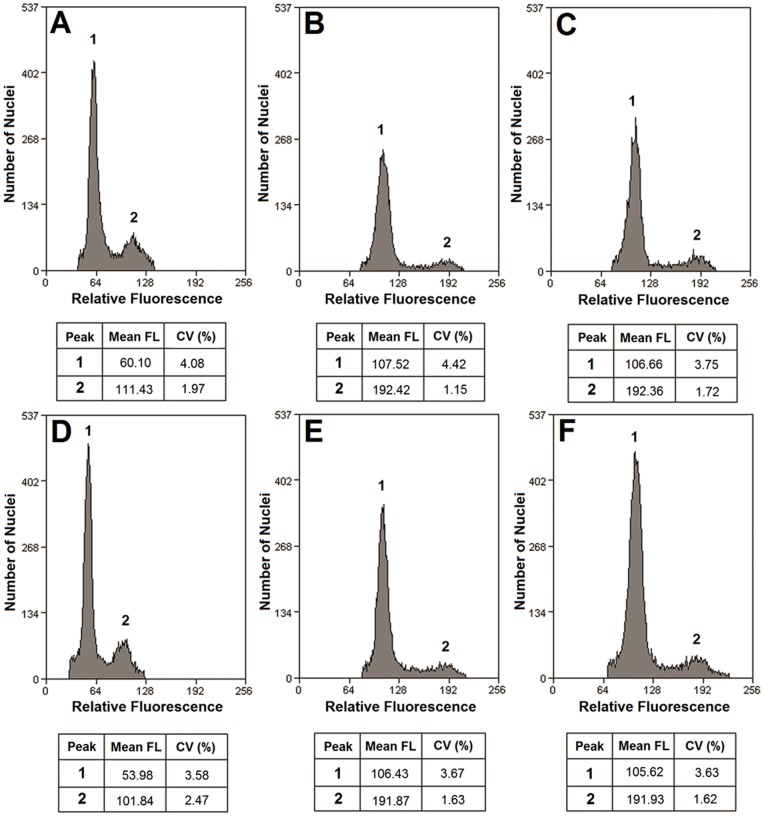
Flow cytometric ploidy analysis of nuclear DNA content of nuclei released by fresh leaf tissue. (A) *S. arvensis* (France-1), (B) *B. napus* (Canada), (C) *B. juncea* (Xining, China), (D) ZS-5, (E) ZS-11, and (F) ZS-13. The peaks marked 1 and 2 indicate nuclei of DAPI-stained nuclei at G_1_-phase and G_2_-phase. The mean channel number (mean fluorescence light (FL)) and coefficient of variation value (CV, %) of each peak were also given.

To estimate plant ploidy by flow cytometry (FCM), we chose two of the wild *Brassica* collections containing A and B-genome-specific PCR products (ZS-11 and ZS-13), and one containing the S-genome (ZS-5). Analysis of nuclei isolated from a leaf yielded a histogram of the longitudinal and abscissa axes, corresponding to the mean of relative number of nuclei of cells and relative fluorescence channel value, respectively. The peak positions represented the ploidy of samples in comparison with the reference standards, and identified two distinct peaks (mean CV = 1.15–4.42%). The reference standards obtained enabled a clear assignment of the ploidy level (Fig. 4A, B, and C). The mean FLs of the G_1_-phase peak positions of ZS-5 and *S. arvensis* were 53.98 and 60.10, respectively and the DNA ploidy of ZS-5 proved to be similar to *S. arvensis* (diploid) (Fig. 4A and D). The ploidy levels of ZS-11 (mean FL = 106.43) and ZS-13 (mean FL = 105.62) were tetraploid, similar to *B. juncea* (mean FL = 106.66) and *B. napus* (mean FL = 107.52) (Fig. 4B, C, E, and F).

### Hybridization Compatibility

Varied hybridization compatibility was found between the three wild accession numbers (ZS-5, ZS-11 and ZS-13) and the three known species controls (*B. nigra*, *B. juncea* and *S. arvensis*). The percentage of pod setting (%) of the crossing of *S. arvensis* with ZS-5 was 33.27%, while those with ZS-11 and ZS-13 were both zero. The sexual compatibility index between *S. arvensis* and ZS-5 was 1.97. The percentage of pod setting (%) of crossing *B. juncea* with ZS-11 (32%) or ZS-13 (22.78%) was higher than with ZS-5 (0.26%). The sexual compatibility index of hybridization between *B. juncea* and ZS-11 and ZS-13 was 2.39 and 1.71, respectively (Table 2). Together with observations of SCM pattern, DNA marker identification and FCM evaluation, these data suggested that ZS-11 and ZS-13 could be *B. juncea*, and that ZS-5 could be *S. arvensis*. The results agreed with the above observations of SCM pattern, DNA marker identification and FCM evaluation.

**Table 2:** Hand-crossing of wild collections by pollens of *Sinapis avensis* (France-1), *Brassica juncea* (Xining, China) and *B. nigra* (Australia), respectively.

Pollen donor	Maternalparent	Number ofmaternal plant	Mean numberof flowers	Mean numberof pods	Percentage ofpod setting (%)	Mean number ofseeds per pod	Sexual compatibilityindex
***S. arvensis***	ZS-5	11	69.18±18.43^ab^	21.64±7.13^b^	33.27±14.37^b^	5.92±1.04^c^	1.97±0.88^b^
	ZS-11	10	49.30±18.27^a^	0±0^a^	0±0^a^	0±0^a^	0±0^a^
	ZS-13	12	57.67±17.97^a^	0±0^a^	0±0^a^	0±0^a^	0±0^a^
	*S. arvensis*	3	96.33±48.34^b^	27.67±21.46^b^	40.41±34.95^b^	3.61±0.24^b^	1.51±1.32^b^
***B. juncea***	ZS-5	8	89.38±19.60^b^	0.25±0.46^a^	0.26±0.47^a^	0.13±0.35^a^	0.001±0.004^a^
	ZS-11	8	59.38±15.79^a^	18.63±7.07^bc^	32.00±11.80^b^	7.25±1.59^b^	2.39±1.23^b^
	ZS-13	8	63.63±20.65^ab^	14.50±8.12^b^	22.78±7.65^b^	7.42±1.38^b^	1.71±0.66^ab^
	*B. juncea*	6	51.33±12.72^a^	26.67±4.27^c^	55.39±19.35^c^	9.06±2.99^b^	5.19±3.06^c^
***B. nigra***	ZS-5	5	58.8±11.00^a^	0.40±0.89	0.59±1.32	0.40±0.89	0.01±0.03
	ZS-11	5	35.60±8.68^a^	0.40±0.89	1.38±3.08	1.20±2.68	0.08±0.19
	ZS-13	4	29.50±11.82^a^	3.25±6.50	7.39±14.77	0.25±0.50	0.07±0.15
	*B. nigra*	3	117.33±23.46^b^	7.00±1.73	6.37±3.06	2.11±0.86	0.14±0.09

Note: Different letters in the same column indicate significant differences (P<0.05) (ANOVA, Scheffe’s test).

Whereas no hybrid seed was formed from the crossing of *B. juncea* (Xining, China) as the paternal parent and *S. arvensis* as the maternal parent, few hybrid seeds were formed from the crossing of *S. arvensis* (France-1) as the paternal parent and *B. juncea* as the maternal parent (Xining, China) (sexual compatibility index = 0.91) (Table 3). The results suggested a low sexually compatibility between *B. juncea* and *S. arvensis.* The percentage of pod setting (%) and sexual compatibility index from backcrossing F_1_ with *S. arvensis* (4.43%,0.13) was lower than backcrossing with *B. juncea* (24.18%,1.87) (Table 3). Hybrid was more sexually compatible with the maternal *B. juncea* than the paternal *S. arvensis*.

**Table 3:** Hand-crossing of *Brassica juncea* (Xining, China) with *Sinapis arvensis* (France-1) and the backcrossing of hybrids to their parents.

Pollendonor	Maternalparent	Number ofmaternal plant	Mean number ofpollinated flowers	Mean numberof pods	Percentage ofpod setting (%)	Mean number ofseeds per pod	Sexual compatibilityindex
***S. arvensis***	*B. juncea*	4	35.75±26.29	4.75±6.29	12.58±15.63	3.21±4.50	0.91±1.69
***B. juncea***	*S. arvensis*	3	41.00±3.61	–	–	–	–
***B. juncea***	F1	9	44.11±28.56	9.22±10.37	24.18±16.62	8.09±4.18	1.87±1.24
***S. arvensis***	F1	10	52.10±17.32	2.10±0.99	4.43±1.93	3.43±2.44	0.13±0.09

(A) ZS-4 × *S. arvensis* (France-1), (B) ZS-5 × *S. arvensis*, (C) ZS-6 × *S. arvensis*, (D) ZS-8 × *S. arvensis*, (E) ZS-9 × *S. arvensis*, (F) ZS-10 × *S. arvensis*, (G) ZS-14 × *S. arvensis*, (H) ZS-15 × *S. arvensis*, (I) ZS-16 × *S. arvensis*, (J) XJ-11 × *S. arvensis*, (K) XJ-Baicheng × *S. arvensis*, (L) *B. juncea* × *S. arvensis*, (M) *B. juncea* × ZS-5, (N) ZS-4 × *B. nigra* (Australia), (O) ZS-8 × *B. nigra*, (P) ZS-9 × *B. nigra*, (Q) ZS-10 × *B. nigra*, (R) ZS-11 × *B. nigra*, (S) ZS-13 × *B. nigra*, (T) ZS-15 ×*B. nigra*, (U) ZS-16 × *B. nigra*, (V) XJ-11 × *B. nigra*, (W) XJ-14 × *B. nigra*, (X) XJ-Baicheng × *B. nigra*, (Y) *B. juncea* × *B. nigra*, (Z) ZS-4 × *B. juncea* (Xining, China), (AA) ZS-5 × *B. juncea*, (BB) ZS-9 × *B. juncea*, (CC) ZS-11 × *B. juncea*, (DD) ZS-13 × *B. juncea*, (EE) ZS-14 × *B. juncea*, (FF) XJ-Baicheng × *B. juncea*, and (GG) *B. napus* × *B. juncea* (♀×♂).

## Discussion

Seed coat development is a complex process in which the ovule differentiates into outer and inner integuments to develop into the seed coat in concert with embryogenesis [13]. The inner integument may give rise to a tegmen and the outer integument to a testa [25]. While the seed coat is primarily derived from the integument palisade of the mother tissue, certain seed coat patterns in amphidiploids exhibit intermediate patterns between the two putative parents, while others resemble only one of the two parents [8]. It is possible that expression of a SCM trait such as seed coat color is controlled mainly by the maternal genotype, but is also influenced by the interplay between the maternal and endosperm and/or embryonic genotypes [26], [27]. At present, some seed coat-related genes have been identified (e.g. Datla and Haughn [28]), however the global genetic program associated with seed coat development has not been completely elucidated. The results of this current study represent a genetic approach to initiate the investigation of the gaps in our current knowledge in this area.

Seed coat patterns have been found to be species-specific in *Brassica* and related species; however, they can vary markedly in nature. For example, researchers had found that three accessions of *B. nigra* had a reticulate pattern, reticulum, among which one accession had a wide and highly undulated reticulum wall with undulations traversing the interspace and the reticulum interspace of the other two accessions contained smaller daughter reticulations [10]. While we discovered variations in *B. nigra* and *B. juncea*, the basic patterns were still reticulate. *B. carinata*, another B-genome species within the U-triangle of *Brassica*, has a SCM pattern resembling *B. juncea* and *B. nigra* [16]. An exception was suggested, however, in the case of an individual accession of *B. carinata* [10].

It was found that developing seeds often exhibit different phenotypes by observing SCM patterns throughout the seed coat developmental process [8]. For instance, there were nine types of seed coat during the seed development of *B. juncea*. These results indicate that parameters for species identification should include the seed coat pattern of the mature seed. Moreover, certain patterns appearing in common among different species during seed development could provide a new way to determine the proximity of their relationships [8].

At present, seed coat patterns have been used for various purposes, including solving classification problems, establishing evolutionary relationships, elucidating the adaptive significance of the seed coat, and serving as genetic markers for the identification of genotypes in segregating hybrid progenies [8], [29], [30]. In our current study, the S-genomic species invariably displayed a rugose type of SCM and the species containing B-genome specific markers always contained a reticulate type of SCM. This phenomenon was found even in the hybrid seeds set on the S-genomic or B-genomic maternal plants. The characteristic of SCM was found to be related to the genome types and was inheritable in the interspecies hybrid progenies formed between *Brassica* species, implying that the characteristic of seed coat pattern is related to genomic type and is probably determined by maternal effect.

According to the morphological and cytological (2n = 16) characteristic, Wang et al. suggested that some wild *Brassica* distributed in Xinjiang and Northwest part of China were *B. nigra* [17]. However, other researchers held the view that wild *Brassica* distributed in Xinjiang was *S. arvensis* (SS, 2n = 18) (e.g. Guan [15]). Compared to the A and C genomes within the *Brassica* genus, B genome is most homogenous to S genome and eight chromosomes in *S. arvensis* (n = 9) have significant homology with that of *B. nigra* (n = 8) [31]. It has been speculated that *S. arvensis* could be the ancestor of *B. nigra* [2]. A report had suggested that Xinjiang wild *Brassica* lost one pair of chromosome and evolved to *B. nigra* and had tried to confirm this evolutionary trend using biochemical classification and molecular markers [32].

*S. arvensis* has been used for breeding in order to cultivate more desirable characteristics [33], [34], [35], including improved resistance to insects and diseases, low erucic acid and sulfuric glycoside levels, male sterility cytoplasm, and containing anti-sclerotinia and anti-split genes, among others [36]. However, *S. arvensis* has been characterized as a “poisonous weed” [2], whose developed root system and strong growth potential, compete for nutrition and water with cultivated crops, leading to reduction of crop production. Due to its small seeds, which are easy mingled during cultivation of crops seeds, and which can survive up to 60 years in the soil, eradication of this weed is difficult [37]. While this species is also treated as weed in China, efforts should be made to evaluate their potential value in breeding before eliminating them completely.

*Brassicas* raise many taxonomic and evolutionary questions, and to address these questions, attempts have been made to study the species relationships using morphological, cytogenetic and biochemical approaches. Due to the similar morphology of the plants, the taxonomies of *B. nigra* and *S. arvensis* are easily confused and to date the issue of wild rape collections in China [38] has not been resolved. Since both *B. nigra* and *S. arvensis* yield the same DNA amplification products for certain molecular markers (e.g. Pankin and Khavkin [23]), differences in SCM patterns could serve as a way to distinguish the two species from each other. This study provides a taxonomic basis for distinguishing between B-genome wild *Brassica* species and *S. arvensis*.

Combined with DNA amplification evidence, FCM and SCM pattern, hand-crossing hybridization can lead to conclusive species identification. Although sexual compatibility may vary with crossing direction and various geographic populations, results of crossing result could provide a basic perspective in this study. Most of the wild accession numbers collected in Xinjiang in this study have similar SCM and relatively high sexual compatibility to *S. arvensis,* and could well be *S. arvensis*. The wild collection XJ-Baicheng had been proved to be tetraploid by FCM evaluation and fully sexual-compatible with *B. juncea* [39]. Together with ZS-11 and ZS-13, the three tetraploid accession numbers, bearing both A- and B-genomic markers, have similar SCM pattern to and could be wild or weedy *B. juncea*. The finding of tetraploid collections as well as those of diploid ones in Xinjiang could provide helpful information to rapeseed breeders. It is worth mentioning that XJ-Baicheng seeds had 100% germination, large seed size, and a yellow seed coat (data not shown). We speculate that the wild collection XJ-Baicheng is a feral plant that escaped from cultivated *B. juncea,* which also yields large yellow seeds.

Although the expression of the seed coat trait is complicated by multiple-gene inheritance, maternal effect and environmental factors [40], the difference in SCM between B-genome species (*B. juncea* and *B. nigra*) and *S. arvensis* seems quite stable. In addition, the SCM patterns we identified here in *B. juncea*, *B. nigra* and *S. arvensis* are congruent with previous reports [8], [10], [11], suggesting that SCM pattern is consistent and reflects the trait of maternal plants, which may be useful in species identification for wild *Brassica* and related species.

## Supporting Information

Figure S1 **Micrographs of hybrid seed coat at low and high magnification.** (A) ZS-4 × *S. arvensis* (France-1), (B) ZS-5 × *S. arvensis*, (C) ZS-6 × *S. arvensis*, (D) ZS-8 × *S. arvensis*, (E) ZS-9 × *S. arvensis*, (F) ZS-10 × *S. arvensis*, (G) ZS-14 × *S. arvensis*, (H) ZS-15 × *S. arvensis*, (I) ZS-16 × *S. arvensis*, (J) XJ-11 × *S. arvensis*, (K) XJ-Baicheng × *S. arvensis*, (L) *B. juncea* × *S. arvensis*, (M) *B. juncea* × ZS-5, (N) ZS-4 × *B. nigra* (Australia), (O) ZS-8 × *B. nigra*, (P) ZS-9 × *B. nigra*, (Q) ZS-10 × *B. nigra*, (R) ZS-11 × *B. nigra*, (S) ZS-13 × *B. nigra*, (T) ZS-15 ×*B. nigra*, (U) ZS-16 × *B. nigra*, (V) XJ-11 × *B. nigra*, (W) XJ-14 × *B. nigra*, (X) XJ-Baicheng × *B. nigra*, (Y) *B. juncea* × *B. nigra*, (Z) ZS-4 × *B. juncea* (Xining, China), (AA) ZS-5 × *B. juncea*, (BB) ZS-9 × *B. juncea*, (CC) ZS-11 × *B. juncea*, (DD) ZS-13 × *B. juncea*, (EE) ZS-14 × *B. juncea*, (FF) XJ-Baicheng × *B. juncea*, and (GG) *B. napus* × *B. juncea* (♀×♂).
(TIF)

Figure S2 **Flow cytometric ploidy analysis of nuclear DNA content of nuclei released by fresh leaf tissue in** ***B. juncea*** **(Xining, China),** ***S. arvensis*** **(France-1) and their hybrid F_1_.**
(TIF)

Table S1 **Genomic specific molecular markers used to test wild rape collected in Xinjiang, China.**
(DOCX)
