# Supplementary figures and images for: Seed Coat Microsculpturing Is Related to Genomic Components in Wild Brassica juncea and Sinapis arvensis
Source: PLoS One. 2013 Dec 30;8(12):e83634. doi: 10.1371/journal.pone.0083634 (PMC3875484; doi:10.1371/journal.pone.0083634)

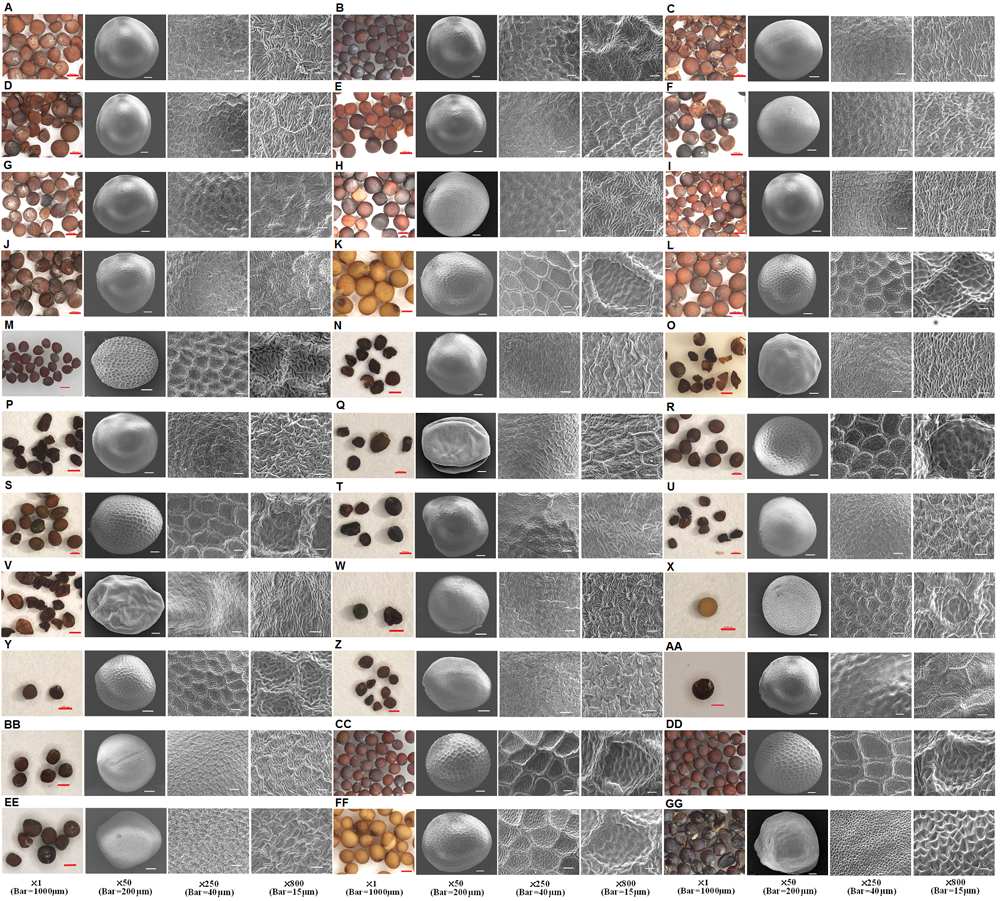

Supplement: Figure S1 — Micrographs of hybrid seed coat at low and high magnification. (A) ZS-4 × S. arvensis (France-1), (B) ZS-5 × S. arvensis, (C) ZS-6 × S. arvensis, (D) ZS-8 × S. arvensis, (E) ZS-9 × S. arvensis, (F) ZS-10 × S. arvensis, (G) ZS-14 × S. arvensis, (H) ZS-15 × S. arvensis, (I) ZS-16 × S. arvensis, (J) XJ-11 × S. arvensis, (K) XJ-Baicheng × S. arvensis, (L) B. juncea × S. arvensis, (M) B. juncea × ZS-5, (N) ZS-4 × B. nigra (Australia), (O) ZS-8 × B. nigra, (P) ZS-9 × B. nigra, (Q) ZS-10 × B. nigra, (R) ZS-11 × B. nigra, (S) ZS-13 × B. nigra, (T) ZS-15 ×B. nigra, (U) ZS-16 × B. nigra, (V) XJ-11 × B. nigra, (W) XJ-14 × B. nigra, (X) XJ-Baicheng × B. nigra, (Y) B. juncea × B. nigra, (Z) ZS-4 × B. juncea (Xining, China), (AA) ZS-5 × B. juncea, (BB) ZS-9 × B. juncea, (CC) ZS-11 × B. juncea, (DD) ZS-13 × B. juncea, (EE) ZS-14 × B. juncea, (FF) XJ-Baicheng × B. juncea, and (GG) B. napus × B. juncea (♀×♂). (TIF) [file pone.0083634.s001.tif]

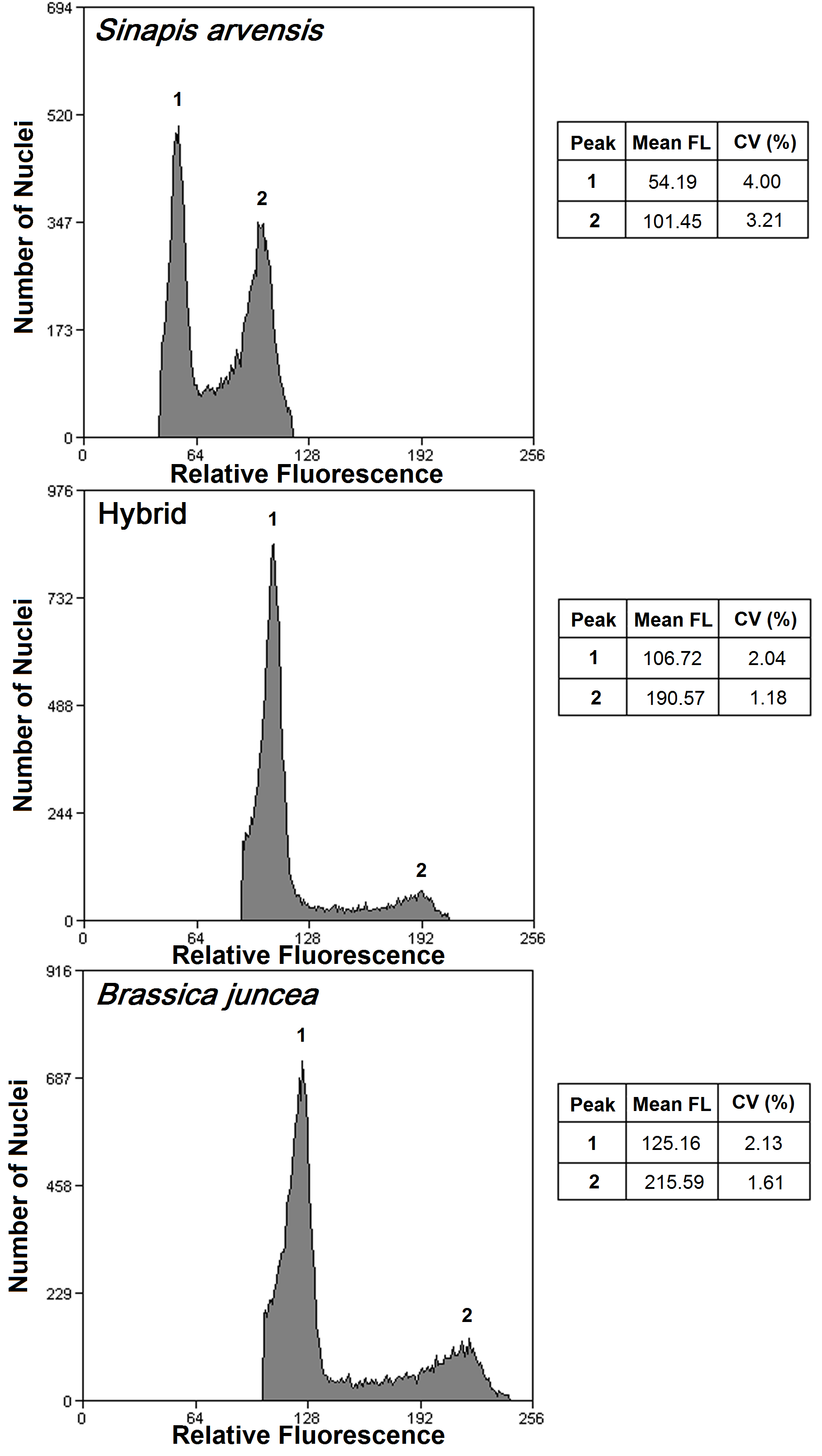

Supplement: Figure S2 — Flow cytometric ploidy analysis of nuclear DNA content of nuclei released by fresh leaf tissue in B. juncea (Xining, China), S. arvensis (France-1) and their hybrid F1. (TIF) [file pone.0083634.s002.tif]
