# Supplementary material for: Seed Coat Microsculpturing Is Related to Genomic Components in Wild Brassica juncea and Sinapis arvensis
Source: PLoS One. 2013 Dec 30;8(12):e83634. doi: 10.1371/journal.pone.0083634 (PMC3875484; doi:10.1371/journal.pone.0083634)
Supplement: Table S1 — Genomic specific molecular markers used to test wild rape collected in Xinjiang, China. (DOCX) [file pone.0083634.s003.docx]

**Table S1. Genomic specific molecular markers used to test wild rape collected in Xinjiang, China.**

| Genome | Primer | Sequence | Size, Ta (℃), cycling | Reference |
| --- | --- | --- | --- | --- |
| A | Na10-B01 | 5’- CAAGTGTCTGCTAGGTGGGG-3’  5’-TCGATCGAAGAAACCAGACC-3’ | 338, 58, A | [18] |
| A , B | Na10-D09 | 5’- GTCGGGTTTGAGTGAGTTGG-3’  5’-CATCGCAGATCCTTCTCTCC-3’ | 280, 60, A | [18,19] |
| B | Ni2-E04 | 5’-TTGCTGAAGACGAGACAACG-3’  5’-TTATGTTCGTTTCCGGTTCG-3’ | 147, 56, A | [18,19] |
| B | pBNBH35 | 5’-GGCATCTGAAGAGAGAGTCCCTTTG-3’  5’-ATCTTCTTCTTGCCATGAGTGGCC-3’ | 329,66,A | [22] |
| C | BN83B1 | 5’-GCCTTTCTTCACAACTGATAGCTAA-3’  5’-TCAGGTTGCCTCGTTGAGTTC-3’ | 194, 56, A | [20-21] |
| C | Na12-C08 | 5’-[GCAAACGATTTGTTTACCCG](http://brassica.nbi.ac.uk/cgi-bin/ace/generic/tree/BrassicaDB?name=GCAAACGATTTGTTTACCCG&class=Primer_info) -3’  5’-[CGTGTAGGGTGATCTAGATGGG](http://brassica.nbi.ac.uk/cgi-bin/ace/generic/tree/BrassicaDB?name=CGTGTAGGGTGATCTAGATGGG&class=Primer_info) -3’ | 333, 56, A | [18] |
| B , S | COL1.1 | 5’-GTTCTAGTATAGATAAGCGATTCACAGGTC-3’  5’-AATGAAGGAACAATCCCATATCCTGTGTC-3’ | 550, 68, A | [23] |
| B , S | SLR1.1 | 5’-CATGCTCGAAACTCATACAATACGG-3’  5’-GAACGAAGCCTTTGATACAGTTACACG-3’ | 386, 68, B | [23] |
| B , S | LFYa.5 | 5’-TAAGATCCAGCGTCCTCTAGCATTAGATTCAA-3’  5’-CACAGAGCTCATGGATTCTGTACTATGGAGA-3’ | 300, 68, A | [23] |

For each marker shown are: marker name; forward (F) and reverse (R) primer sequences; approximate size and variation determined against molecular weight marker in agarose gel; optimized annealing temperatures (Ta);cycling profiles“ A ”(94°C for 1 min, Ta for 1 min, 72°C for 1min) and “ B” (94°C for 1 min,Ta for 30s,72°C for 1min)
